# Telephonic verbal autopsies among adults in South Africa: a feasibility and acceptability pilot study

**DOI:** 10.1136/bmjopen-2024-090708

**Published:** 2025-02-19

**Authors:** Carmen Sant Fruchtman, Ian Neethling, Debbie Bradshaw, Daniel Cobos Muñoz, Diane Morof, Sizzy Ngobeni, Xolani Ngwenya, Anita Edwards, Tracy Glass, Kathleen Kahn, Kobus Herbst, Erna Morden, Nesbert Zinyakatira, Pamela Groenewald

**Affiliations:** 1 Swiss Tropical and Public Health Institute, Basel, Switzerland; 2 University of Basel, Basel, Switzerland; 3 Burden of Disease Research Unit, South African Medical Research Council, Cape Town, South Africa; 4 University of Greenwich, London, UK; 5 School of Public Health and Family Medicine, University of Cape Town, Cape Town, South Africa; 6 Epidemiology and Public Health, Schweizerisches Tropen- und Public Health-Institut, Basel, Switzerland; 7 Division of Global HIV and Tuberculosis, US Centers for Disease Control and Prevention, Durban, South Africa; 8 US Public Health Service Commissioned Corps, Rockville, Maryland, USA; 9 MRC/WITS-Agincourt Rural Public Health and Health Transitions Research Unit, University of the Witwatersrand School of Public Health, Johannesburg, South Africa; 10 Africa Health Research Institute, Durban, South Africa; 11 Social Science, Africa Health Research Institute, Mtubatuba, South Africa; 12 University of the Witwatersrand School of Public Health, Johannesburg, South Africa; 13 DSI-MRC South African Population Research Infrastructure Network, Medical Research Council of South Africa, Tygerberg, South Africa; 14 Health Intelligence, Western Cape Government Department of Health, Cape Town, South Africa

**Keywords:** Telemedicine, Health Services, Health policy, Mortality

## Abstract

**Abstract:**

**Objective:**

This pilot study explores the feasibility and acceptability of using telephonic verbal autopsies (teleVAs) in South Africa to collect information on causes of death.

**Design:**

Quantitative and qualitative data collection methods were used to evaluate the feasibility and acceptability of these telephonic interviews.

**Setting:**

The teleVA pilot was conducted in South Africa’s Western Cape province. The qualitative component also included two rural South African Population Research Infrastructure Network nodes (Africa Health Research Institute in KwaZulu-Natal and Agincourt in Mpumalanga), which had transitioned to teleVAs during COVID-19, allowing exploration of teleVA’s feasibility in both urban and rural settings.

**Participants:**

We recruited 229 respondents to participate in a pilot teleVA. After each VA, VA interviewers filled in a survey to assess their perceptions and discern if they experienced any technical challenges. We also conducted 18 in-depth interviews with both interviewers (n=6) and respondents (n=12) to explore their views on the acceptability of the teleVA. We conducted a thematic analysis of these interviews.

**Interventions:**

VA was piloted over the phone, instead of face-to-face.

**Primary and secondary outcome measures:**

Primary outcomes focused on the feasibility and acceptability of phone VAs among both interviewers and respondents. Secondary outcomes evaluated the quality of teleVAs.

**Results:**

Participants expressed willingness to participate in teleVAs, considering them valuable for public health planning and decision-making. The feasibility of collecting next-of-kin information proved challenging, with incomplete or incorrect contact details posing future logistic issues. Only one question out of 76, showed a statistically significant difference in the proportions of non-informative teleVA compared with face-to-face VA.

**Conclusions:**

The study offers valuable insights into using teleVAs to gather cause of death information in resource-limited settings. It highlights the feasibility and acceptability of teleVAs while emphasising the need for comprehensive planning, integration with the civil registration and vital statistics system and community participation enhancement.

**STRENGTHS AND LIMITATIONS OF THIS STUDY**

- This study is among the first to evaluate telephonic verbal autopsy (teleVA) as an alternative to traditional face-to-face VAs.
- A key strength lies in its quantitative and qualitative examination of teleVA, including perceptions from interviewees and interviewers, as well as a quality comparison between teleVA and face-to-face VAs.
- The inclusion of South African Population Research Infrastructure Network nodes provided an opportunity to explore teleVA in both rural and urban contexts and to compare it with traditional VAs.
- A limitation is the timing of the study which was conducted after the initial COVID-19 peak; this may have influenced respondents’ openness to teleVA as they became more accustomed to remote interactions.
- Although the findings support teleVA as a feasible and promising alternative with implications for research, practice and policy, further research is recommended, particularly involving technological assessments and perspectives from decision-makers.

## Background

 The importance of valid cause-specific mortality statistics to inform health priorities and policy is unequivocal.^1^ Civil registration and vital statistics (CRVS) systems should provide reliable and universal mortality and cause of death (CoD) statistics which are essential for policymaking and resource allocation. However, mortality information systems in many countries still do not achieve good coverage and good quality.^2^ In the absence of robust CRVS systems, verbal autopsy (VA) methods have emerged as a tool to infer the probable causes of death at the population level.^3^ A VA is a structured interview with relatives or a close caregiver of the deceased, which aims to obtain information on the clinical symptoms, signs and events during the illness that ultimately led to the demise of the decedent.^3^ The interview is then followed by a review of the VA information to assign a CoD by either a clinician or using computerised software.^4^

Since the 1970s, VAs have been used to assign the CoD for deaths occurring outside health facilities. The VA tools have been improved and standardised globally during the last 15 years.^5^ The implementation of VA into routine CRVS systems has presented an opportunity to fill the gap in information on CoD for deaths, especially those occurring in the community.^6^ Studies have validated the accuracy of VA at the population level by comparing its results to medical records, demonstrating its ability to generate meaningful mortality statistics that are crucial for public health policymaking.^7^,^9^

However, VA implementation is resource-intensive, primarily due to personnel costs driven by interviewer time for contacting respondents and travelling to communities.^10^,^12^ Despite VA interviews typically lasting 30–40 min, a study on a Tanzanian VA pilot revealed that interviewers spent an average of 42 hours gathering information about next-of-kin (NoK) and 47 hours on travel per VA.^13^ This prolonged process was attributed to an inefficient death notification system and multiple trips to remote areas, imposing a significant burden on already strained healthcare professionals and hindering the scalability and integration of VA into routine mortality information systems.^6^

South Africa has made considerable progress in strengthening its CRVS system and reaching almost 90% death registration coverage for individuals older than 2 years.^14^ However, a key challenge remains: more than half of all deaths occur outside of health facilities, making it difficult to capture all death registrations.^15 16^ In addition, the data collection and accompanying processes for CRVS are lengthy. Local-level health services do not have timely access to information about the deaths in their district, which is critical to inform health planning and response, especially during an epidemic.^17^ The current process makes it impossible for such information to contribute meaningfully to planning interventions or monitoring the quality of care. In addition, a review of official CoD data reveals ongoing concerns about data validity.^18^,^21^

VA has been used in health and demographic surveillance sites (HDSS) in South Africa for many years to assign a CoD to all deaths occurring in those communities.^22 23^ However, these sites only cover a small percentage of the population.^22^

In VA systems, in general, historically all records were assigned a CoD by a physician who reviewed the narrative and responses to the questions.^24^ More recently, automated coding software has been developed to assign causes of death, making the process substantially quicker and less costly.^20^ This has enabled consideration to be given for routine implementation of VA within a CRVS. The South African National Cause-of-Death Validation study (NCoDV) conducted VAs for a national sample of deaths to provide empirical data for correcting cause-specific mortality fractions from official CRVS data (see further details below^25^).

Following the NCoDV study, we aimed to further the understanding and possibilities of routine implementation of VA in South Africa by using telephonic VAs (teleVAs) to collect information on the decedents CoD that occurred outside a health facility. Key barriers to routine implementation of VA are the resources needed for staff, training of staff, travel, tablets and supervision.^6^ We therefore aimed to explore and assess the acceptability and feasibility of conducting teleVAs which could substantially reduce the time and costs associated with the method. In addition, we compared the VA response patterns by examining the percentage of valid responses (yes or no) to investigate whether teleVA produces similar quality information compared with face-to-face interviews from the NCDoV study.

We had previously conducted a teleVA study in Tanzania, which showed promising results, further motivating our interest in exploring the potential of teleVAs for routine implementation in resource-limited settings like South Africa.^26^ Additionally, at the time of this study, non-pharmaceutical interventions had been implemented in South Africa to prevent transmission of COVID-19 and researchers at demographic and health surveillance sites in South Africa were also exploring conducting teleVAs rather than face-to-face VA interviews to comply with pandemic restrictions.

## Methods

### Study design

We undertook a pilot study to explore the feasibility and acceptability of teleVAs.^27^ Feasibility refers to the practicality of implementing an intervention, including the extent to which its components and activities can be carried out effectively, considering factors such as the delivery methods used. Acceptability, on the other hand, relates to how suitable and appropriate the intervention is perceived to be by both patients and healthcare professionals, as well as its alignment with daily life practices.^28^ Over a 2-month period (September 2021 and October 2021), we conducted teleVAs with NoK contacts of deceased individuals, recording data via interviewers’ call log sheets. We also conducted surveys to assess the quality, acceptability and feasibility of each teleVA, followed by in-depth interviews (IDIs) with both respondents and interviewers after the pilot to gain further insights into their perceptions of conducting teleVAs.

This pilot was embedded within the larger Strengthening Mortality Surveillance (SMS) study, which was developed to respond to a gap in the district health information system regarding access to timeous and accurate CoD data during the COVID-19 epidemic. There were several subprojects within the SMS which included a rapid assessment of CoD data collection and public health use in South Africa, the teleVA pilot and a scoping study of online capture of the medical certificate of CoD in South Africa.^29^

### Study setting

The teleVA pilot was conducted in the Cape Town metro which falls under the Western Cape Provincial and the City of Cape Town Health Departments. Furthermore, two nodes of the South African Population Research Infrastructure Network (SAPRIN) HDSS (Africa Health Research Institute (AHRI) and Agincourt)) were included in the qualitative part of the study.^22^ The AHRI node is located in rural KwaZulu-Natal and Agincourt is located in the rural province of Mpumalanga. As a result of the COVID epidemic, both nodes had shifted their routine in-person VA activities to be conducted over the phone starting in 2021. This enabled us to explore the opportunities and barriers for teleVA in both metropolitan and more rural settings in South Africa.

### Intervention: teleVA pilot

Six interviewers experienced in conducting face-to-face VAs were recruited for this teleVA pilot study. Interviewers were provided with 5 days of refresher VA training as well as training on conducting teleVAs. The teleVAs were conducted over a 7-week period between August 2021 and October 2021. The V.1.5.3 of the electronic (Open Data Kit) 2016 WHO VA instrument was used, incorporating the COVID-19 package.^30^ A data system for electronic data capture and storage was set up using KoboToolbox which was installed on the Western Cape Provincial Health Data Centre (WCPHDC) server.^31^

TeleVA respondents (NoK) were eligible if they were aged 18 years or older and if their deceased family member was (1) aged 18 years or older, (2) their death occurred outside a health facility and (3) the CoD was not due to an unnatural cause (eg, an accident or suicide).

We used three different methods to identify deaths that occurred outside a health facility with contact details for NoK for study recruitment and included:

1. Out-of-facility deaths were retrospectively identified through linkage of known COVID-19, tuberculosis, HIV and diabetes cases from the WCPHDC with the National Population Register data held by the South African Medical Research Council (SAMRC). These linkages were restricted to cases with South African identity numbers. Contact information for relatives was sought from other available data sets in the data centre and those with information for relatives were selected (n=3977).
2. Emergency medical services (EMS) identified deaths outside a health facility to which they attended during August 2021 and obtained contact details for the person who called EMS at the time of the death (n=358).
3. Funeral parlours (FPs) were approached to assist with identifying out-of-facility deaths and obtaining NoK contact details and their permission for project staff to make contact to arrange an interview (n=7).

**Figure 1:**
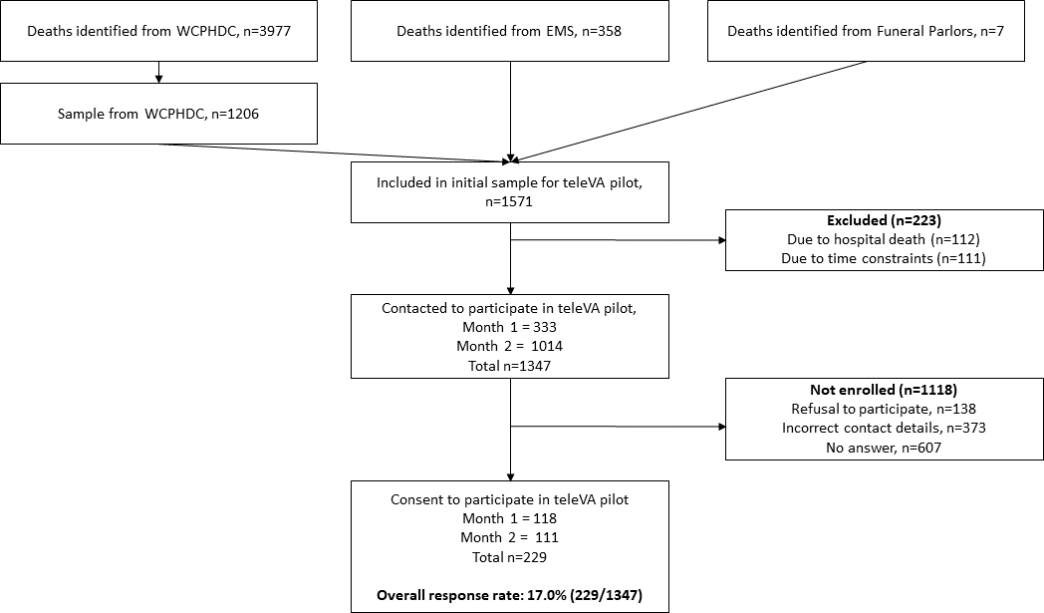
Flow diagram of respondent sample identified, contacted and consented to teleVA interview. Strengthening Mortality Surveillance (SMS) for improved monitoring of HIV/AIDS, TB and COVID-19 in South Africa, September 2021. EMS, emergency medical services; n, number; TB, tuberculosis; teleVA, telephonic verbal autopsy; WCPHDC, Western Cape Provincial Health Data Centre.

During the period from December 2020 to September 2021, a total of 4342 deaths outside a health facility were identified included contact details for NoK. A convenience non-probability sample of 600 deaths was targeted. We assumed a refusal rate of around 60%, slightly lower than expected for a telephone survey (70–80%) as we felt that respondents might be more likely to respond positively to an interview request coming from the Western Cape Health Department.^32^ Thus, 1571 recent deaths that occurred outside a health facility were selected, ensuring at least a 1-month gap since death had transpired before an interview was attempted (more detail in figure 1). Since the contact information for home deaths identified by the WCPHDC took some time to obtain and the fieldwork time was limited, we included cases as the information became available.

A call centre was established in August 2021 at the Western Cape Department of Health with a team of six trained interviewers. These interviewers used the KoBoToolbox app to capture data while contacting NoK via phone to obtain permission for teleVA interviews and identify the most suitable respondent. Once the NoK agreed to the interview, the interview proceeded immediately, or an appointment was set up. Verbal consent was obtained over the phone and recorded using an Android device. Initially, a maximum of 10 attempts were made to contact each respondent before a final outcome (successfully interviewed, refusal to be interviewed, unable to be contacted) was declared. After the first month, due to fieldwork time constraints, the maximum number of attempted calls was reduced to three per respondent.

NoK were reimbursed with a R100 (∼US$5.4) e-voucher after successful completion of the teleVA and only Western Cape respondents who completed an IDI received a R50 e-voucher. The e-voucher was designed to compensate for the respondent’s time and the inconvenience of participating in the teleVA interview. The vouchers were disbursed through the Flash mobile application (https://flash.co.za/).

A call log was maintained by the teleVA interviewers. Data from the call log described the date, time and outcomes for each individual call, the total number of calls and the final outcome for each potential participant.

### Feasibility and acceptability evaluation

Feasibility was rigorously assessed by analysing response rates and the quality of the entire VA process was assessed by comparing valid responses (‘yes’ or ‘no’) from the teleVAs with face-to-face VAs from the NCoDV Study.^33^ Concurrently, acceptability was evaluated through a comprehensive post-teleVA survey and IDIs conducted with both the respondents and interviewers. This dual approach aimed to shed light on the viability and receptiveness of conducting teleVAs within this context.

### Post-teleVA survey

After each teleVA, the teleVA interviewers filled in a survey to further assess the feasibility of teleVA. The survey captured the following information: (1) the number of calls made to the NoK before successfully conducting an interview, (2) the occurrence of any technical challenges, (3) sound quality, (4) if any interruptions occurred during the call, (5) the level of comfort of the interviewer, (6) the respondent’s attitude as perceived by the interviewer and (7) the overall satisfaction of the interviewer during the call. This survey had been previously implemented in Tanzania.^26^ As this was a pilot study, the level of missing data was documented but no imputation was undertaken.

### Quality of the VA responses

The root (entry level) questions in the VA are closed-ended allowing the selection of one of the following options ‘yes’, ‘no’, ‘don’t know’ or ‘missing’. Questions with responses ‘yes’ or ‘no’ were defined as informative responses, with ‘don’t know’ and ‘missing’ defined as non-informative responses. To assess the quality of the teleVA responses, we compared the proportions of non-informative responses, for each root (entry level) VA question with those for face-to-face VAs conducted during a previous study (NCoDV2017/18).^25^ The difference in the proportions of non-informative responses between teleVA and face-to-face VA was tested for each of the VA root questions using a χ² test.

### Individual in-depth interviews

We conducted semistructured interviews with teleVA respondents as well as interviewers to understand the feasibility and acceptability of teleVA. We used the VA call log to purposively recruit individuals from a diverse group of teleVA respondents for the qualitative interviews. We also recruited VA interviewers from the Western Cape pilot, as well as from the SAPRIN nodes, in order to capture experiences in a different setting and from VA interviewers who had experience in VA and teleVA.

All participants in the study gave informed verbal consent after being sent a written study information sheet via WhatsApp and a verbal explanation of the consenting process at the beginning of the interview. Verbal informed consent was recorded during the phone interviews.

All interviews with Western Cape respondents and interviewers were conducted by CSF (first author). The interview was conducted in English, except for two that were conducted in Xhosa and Afrikaans, respectively, with the support of a translator. In the SAPRIN nodes, SN conducted three interviews in Shangaan (Agincourt) and three were conducted by XN in Zulu (AHRI). The interviews were conducted based on a question guide that had been developed and tested for the study (see the full guide as online supplemental materials). Semistructured interviews were used to contextualise the findings of the survey. Probes were used to elicit additional information based on the course of conversations and the interviewer’s observations.

All interviews were conducted by phone, using MS Teams and audio-recorded, after the interviewee’s consent. Only the interviews conducted by SN and XN were transcribed and translated into English to allow CSF, who led the analysis, to immerse in the data.

The IDIs were analysed using rapid qualitative methods.^34^ The information collected during the interviews was compiled and summarised using a rapid assessment procedure (RAP) sheet by the researcher conducting the interview. The RAP sheet was created using the domains of acceptability, that is, comfort and satisfaction and feasibility, that is, logistics, technical challenges and sound quality which allowed the findings to be categorised using a deductive approach.^35^ This allowed the researchers to immerse in the data and permitted early identification of findings as data were being collected. Thereafter, inductive coding was used to allow for emerging themes to be identified. Representative quotes were extracted from the audio recordings and categorised in the RAP sheet.

### Patient and public involvement

This pilot study actively involved the public by assessing the feasibility and acceptability of teleVAs through next-of-kin in-depth interviews. Their feedback shaped the understanding of teleVA implementation, participant burden and alignment with daily practices, ensuring future developments are guided by public experience.

## Results

From a convenience sample of 1571 deaths, 229 NoK consented to conduct a teleVA, of which 54% were females and 77% were aged 50 years or older. The decedents age ranged from 19 to 98 years, with a mean age of 59 years for males and 62 years for females. Most respondents were close family members, consisting of children (37%), other family (22%), spouses (19%) and siblings (10%). Only 7% were unrelated.

For the qualitative study, we recruited a total of 18 respondents including teleVA respondents and interviewers were conducted. Two respondents who had participated in the SMS study and were contacted for an IDI refused to participate. The sample included 12 teleVA respondents (6 female and 6 male) and 6 teleVA interviewers (3 female and 3 male). In month 1, a total of 1237 calls were made to the 333 respondents approached for consent to a teleVA interview (average of 3.7 calls per respondent (1237/333); maximum of 10 calls per respondent). A total of 118 VAs (35.4% response rate (118/333)) were conducted in month 1 with an average of 2.9 calls needed per consenting respondent to achieve a successful VA. Overall, 9.5 calls to potential participants were required to achieve each successful interview. The average time for each interview was 25 min, ranging between 17 and 68 min.

During month 2, 1014 respondents were approached with a total of 1498 calls conducted, an average of 1.5 calls per respondent. As described in the methods, we limited the maximum call attempts per respondent to three during month 2 due to time constraints. A total of 111 VAs (10.9% response rate (111/1014) was conducted during month 2. Over the whole study period, the response rate was 17.0% (229/1347).

The rate of successful contact varied by source of recruitment, with success rates of 42.9%, 38.7% and 11.3% for participants sourced from FPs, EMS and WCPHDC, respectively. The main reasons for failure to make contact with potential participants were due to unanswered calls (45.1%) and invalid numbers (27.7%), while refusals accounted for 10.2% (table 1).

**Table 1:** Success rate for eligible respondents contacted by source. Strengthening Mortality Surveillance (SMS) for improved monitoring of HIV/AIDS, TB and COVID-19 in South Africa, August–September 2021

Final outcome	Source			Total (%)
	EMS	FP	WCPHDC	
Invalid number	23	1	349	373 (27.7)
No answer	95	1	511	607 (45.1)
Refusal	48	2	88	138 (10.2)
Success	105	3	121	229 (17.0)
Grand total	271	7	1069	1347 (100)
Contact success rate %	38.7	42.9	11.3	17.0

EMSemergency medical servicesFPfuneral parloursTBtuberculosisWCPHDCWestern Cape Provincial Health Data Centre

The majority of teleVAs were conducted without encountering significant technical impediments, accounting for 84.3% of the cases. The remaining 15.7% of teleVAs were, however, confronted with technical challenges. These challenges were classified into several categories, with 11.4% attributable to poor connectivity, 0.9% to respondents’ depleted battery power, 0.4% to respondents exhausting their available airtime and 3.1% to various other technical difficulties. The technical issues were documented in the post-teleVA survey which was completed by the interviewers after completing each interview.

The quality of teleVA information was assessed by comparing the proportion of non-informative VA responses to root questions in teleVA with face-to-face VAs. Only one question, ((Id10166) During the illness that led to death, did (s)he have fast breathing?) showed a significant difference in the proportions of non-informative responses (Pearson χ²=8.745; p<0.01), with teleVA having a higher proportion of non-informative responses than face-to-face VA (5.2% vs 2.2%), see online supplemental table S1.

In addition, only 3% of the causes of death were undetermined according to physician-coded VA.

The information from the IDIs with teleVA interviewers and respondents was coded and categorised into six main themes to explore the acceptability of conducting teleVAs.

### Theme 1: interviewers needed time and training to conduct VA over the phone instead of in-person

In the SAPRIN nodes and the Western Cape, interviewers had prior experience conducting face-to-face VA interviews before the teleVA pilot. However, transitioning to the telephonic format posed a challenge for them. One difficulty they encountered was discussing sensitive topics covered in a VA over the phone. According to several interviewers, expressing condolences for the loss of a family member seemed more effective during in-person interviews but this proved to be more difficult during phone interviews. Interviewers frequently encountered difficulties in finding suitable methods to provide comfort to respondents experiencing distress during the phone interviews.

*It was a bit strange at first, because the questions we ask are very personal. It was very difficult to understand the reactions of the respondents, especially me being a male, asking very personal questions*. ID2, male teleVA interviewer, Western Cape

*Some when they were giving information, they started crying - I didn’t know what to say to the person, because it was on the phone. I stayed quiet and waited, then asked if we should continue.* ID5, male teleVA interviewer Western Cape

In the Western Cape, interviewers encountered an additional challenge related to managing rejection during phone interviews. They observed that some respondents were less respectful on the phone compared with face-to-face interactions with families. Surprisingly, this experience of rejection led to the interviewers forming a closer team bond and providing peer support. This support system enabled them to develop strategies to enhance their approach over time when contacting NoK. As a result, interviewers underscored the importance of thorough and specialised training for conducting effective VA interviews over the phone.

*It’s much more difficult to reject when you’re in person. There’s nothing else you can do if a person hangs up. When they see your face and you will say it won’t take long…, they see this person is at work, so maybe they need help…but over the phone there’s not much that you can do.* ID3, female teleVA interviewer, Western Cape

In contrast, the interviewers from the SAPRIN nodes mentioned the openness of the family members to participate in the teleVA. This could have been due to the communities of the SAPRIN nodes being very familiar with the HDSS work and call centre staff calling for health-related interviews twice a year, versus the ‘cold’ calling in the Western Cape.

### Theme 2: perceptions of the social and individual benefits of participating in a teleVA

In general, the interviewed respondents appreciated the contact of government officials to inquire about the death of a family member. They saw the VA as a mechanism of the Department of Health to improve the health of the population which many perceived as caring about the passing of their family member. Some respondents who lost family members to an unknown cause mentioned that they would have appreciated hearing feedback from the VA, indicating the probable CoD.

*This is awesome to have understanding of what is happening. I appreciated that someone took the time to know we’re ok. Gave me hope in our health system.* ID5, female respondent, Western Cape

In the Western Cape, all participants in the pilot study received a small voucher as compensation for their participation, while in the SAPRIN nodes, there was no such compensation. There were mixed responses regarding the importance of the voucher in the Western Cape. Among the respondents, some mentioned that they appreciated and understood the importance of the VA for public health policy and therefore were happy to participate even without a voucher. Other respondents mentioned that considering the time it took to do the VA, the provision of a voucher was very relevant.

*It was a nice thing to have, but not essential. I would have done it anyway*. ID3, male respondent, Western Cape

The interviewers also provided similar mixed responses. Some had the feeling that without vouchers the response rates would have been lower, while others thought this was not important for the participation rate.

*Sometimes you get the feeling. If we remove the voucher that we give the respondents, the response rate would have gone down.* ID2, male teleVA interviewer, Western Cape

### Theme 3: building trust over the phone to conduct a VA

In all three settings, the interviewers generally described the process of finding the right respondent as a difficult process. The telephone numbers provided or details of the NoK were not always correct. However, those who would pick up the phone would usually help to try to find the right respondent.

An interesting difference between the SAPRIN nodes and Western Cape was building rapport with respondents. In the Western Cape, the interviewers required several attempts of calling and sending information via WhatsApp or SMS before managing to conduct a VA. While in the SAPRIN nodes, this was quite straightforward as community members were used to participating in research and often had previously met the interviewers in person.

*Some people who are young, they understand better talking over the phone, but older people don’t. I think people who are18–40[years old] they are more ok with this. But lots of our respondents are older people, because they’re usually the ones taking care of the deceased.* ID6, male teleVA interviewer, SAPRIN node

### Theme 4: teleVA respondents felt comfortable discussing the VA over the phone

During the interviews, we also explored the level of comfort that the respondents had in discussing the VA topics on the phone. There were mixed opinions on this topic, some respondents mentioned that they preferred doing a VA on the phone as they felt less pushed to participate at the time of the first contact, while others mentioned the importance of discussing these topics face-to-face. Furthermore, several respondents mentioned that due to the COVID-19 situation, they understood why the interviews were done telephonically.

*I was very comfortable doing the interview, but would have preferred in person, to see face expressions. The good thing on phone, I could ask questions and not being judged. But face-to-face is a bit more personal.* ID 1, female respondent, Western Cape

The interviewers also mentioned that despite the initial challenges to discuss sensitive topics on the phone, the teleVAs were often more comfortable as the respondents would limit the expressing of their emotions which was sometimes very challenging to manage during the face-to-face interviews. Lastly, several respondents also mentioned that during the teleVA they felt less shy to ask questions if there was something they would not understand.

*She made me feel very comfortable and that helps. There was no pushiness. She asked and appreciated my time. Gives you the sense of being valued and your time also being important.* ID5, female respondent, Western Cape

In one of the SAPRIN nodes, there was a period where face-to-face and phone interviews were combined as part of the routine surveillance at the node. The interviewer would reach out to respondents on the phone and if they preferred to answer in person, she would travel and conduct an in-person face-to-face interview. She described this as an efficient model, as she could decide to visit those people who felt uncomfortable on the phone or would not pick up when she was calling. This was also the case for elderly people who had some challenges in hearing her speak on the phone.

*Some people don’t hear well over the phone or prefer discussing these topics in person. From 50 participants, maybe 1 prefers home.* ID7, female teleVA interviewer, SAPRIN node

### Theme 5: phone interviews were perceived as a safer and more flexible tool by the interviewers

The interviewers raised several benefits of doing the VAs over the phone, most being related to the fact that the teleVAs were more flexible and less time-consuming than doing the VA in person. Several interviewers, as well as respondents, indicated that they appreciated being flexible about the time of day the interview could be conducted.

*…Cheaper, because you save time, no going to households. Sometime you can go there and the respondent isn’t there. Over the phone you can call another time. Easier to get people.* ID6, male teleVA interviewer, SAPRIN node

*None expected the numbers to grow as quickly as they are. It’s working well. If we were in the field, we wouldn’t be able to do 5 interviews. In theory, we can do more now, but depends how many potential respondents answer.* ID3, female teleVA interviewer, Western Cape

The respondents said it was easier to ask to postpone the interview if it was by phone than in person and they appreciated having less pressure.

Furthermore, the interviewers, as well as some respondents highlighted the safety aspect as an important consideration when choosing between conducting phone versus in-person VAs.

*Some of the people we interview are living in areas that are dangerous. Doing it over the phone is much safer. Once you are out there you are on your own. It’s been positive in that sense*. ID1, male teleVA interviewer, Western Cape

### Theme 6: respondents found emotional support in participating in VA interviews

Several respondents described how participating in the VA process helped them process their trauma and grief. They described that the discussion with the teleVA interviewer was at times very painful, but it also helped them open up and discuss the death with other family members.

*The questions made me very emotional, as we never spoke about this. After the interview, I broke down and cried. After waking up, I talked with my mom about this and we opened up*. ID1, female teleVA respondent, Western Cape

## Discussion

The pilot study demonstrated that teleVAs are generally acceptable to participants who appreciated their potential contribution to public health planning and decision-making. However, feasibility challenges were identified, particularly regarding incomplete or inaccurate NoK contact information. The quality of data collected through teleVAs was largely comparable to face-to-face VAs, with only one of 76 questions showing a significant difference in non-informative responses between the two methods.

The study had two primary objectives: to evaluate the feasibility and acceptability of implementing teleVAs and to compare their data quality to traditional face-to-face VAs. This builds on prior small-scale pilot studies, such as one conducted in Tanzania in 2019 that explored the potential of teleVAs in cost and time savings. This initial pilot, yielded promising outcomes and underscored the absence of significant barriers to teleVA implementation.^26^ Furthermore, the impact of the COVID-19 pandemic in 2020 prompted a transition from face-to-face to teleVA, facilitating data collection during lockdowns in various settings.^36 37^

The pilot in the Western Cape suggests that teleVAs have the potential to improve the efficiency of VA data collection. The acceptability of teleVA for both respondents and interviewers was high and had many advantages such as flexibility, safety, as well as a sense of comfort in discussing sensitive topics on the phone rather than in person. Interestingly, these advantages align with Nasaruddin’s findings in Malaysia, despite the very different contexts.^36^

Surprisingly, some respondents described how engaging in the extensive VA questionnaire helped them and their families process their grief, which contrasts with Hinga’s^38^ findings who reported significant emotional distress among VA respondents due to the face-to-face VA process. Such contrasting experiences may underscore the cultural specificity of VA in different contexts, suggesting that the impact of VA interviews on participants’ emotional well-being can vary based on cultural factors.^39^

The response rate of 17% achieved in our pilot study to recruit teleVA respondents compares favourably with response rates from other telephone surveys which typically range between 5% and 30%.^40^ This is encouraging, given the lack of prior sensitisation in the community and the use of a ‘cold calling’ approach. Nevertheless, it underscores the importance of involving the community in the design and implementation of such processes.^38^ Similar to the National Cause-of-Death Validation study, recruiting NoK for community deaths proved challenging effective implementation of routine VA will require integrated approaches within the CRVS system to ensure timely notification of community deaths for conducting VAs.^6 33^

Our findings also indicate that the success rate of ‘cold-calling’ for teleVA largely depends on the persistence and training of the interviewers. This is evident from the higher success rate of EMS respondents (39%) compared with WCPHDC respondents (11%). The first month of fieldwork targeted mostly cases identified through EMS and multiple call attempts (10) were made to each respondent before giving up. In the second month, interviewers were instructed to make only three call attempts which likely explains the lower success rate during that period. Therefore, the investment in making 10 call attempts was probably justified in comparison to the efficiency of making three attempts. Conversely, the higher response rate from FP respondents might be due to the different recruitment strategies employed which involved immediate contact with NoK.

Additionally, a small-value voucher (less than US$6) was provided to respondents in the Western Cape as a token of appreciation. While this approach was specific to the Western Cape and was not implemented at SAPRIN sites, it may have contributed modestly to response rates. We explored during the interviews and respondents expressed mixed views on the significance of the voucher in their decision to participate. Several participants emphasised their intrinsic motivation, citing a desire to support public health efforts as their primary reason for involvement, rather than the voucher itself.

It is worth noting that the study was conducted during the COVID-19 pandemic, a period when respondents might have been more willing to participate due to heightened awareness of public health efforts. This context may have positively influenced response rates and the perceived value of the teleVA process.

The training of interviewers is critical not only for the successful recruitment of NoK but also for ensuring the quality of VAs. Evidence suggests that well-trained interviewers conducting VAs result in fewer undetermined causes of death.^41^ This aligns with our findings, as the teleVA pilot recorded only 3% of undetermined causes of death, compared with over 15% in our previous face-to-face VA study. This significant improvement underscores the impact of enhanced training and experience in achieving higher-quality VAs. Comprehensive training, coupled with robust monitoring and supervision, remains indispensable for maintaining the quality of VAs.^42^

Specialised training tailored to teleVA interviewers is particularly important for addressing challenges unique to this mode of implementation. During acceptability interviews, interviewers identified specific obstacles, such as handling phone refusals and managing respondents’ grief remotely. Targeted training on these aspects could help overcome these challenges and further improve both the success rates and quality of teleVAs.

Scaling teleVA implementation across the country would require considerable resources for training and monitoring. However, teleVAs offer a valuable opportunity to centralise and standardise interviewer training. By reducing the number of interviewers required and focusing on teleVA-specific skills, this approach could streamline operations and enhance efficiency, making nationwide implementation more feasible.

Lastly, a crucial aspect in comparing teleVA with face-to-face VAs is assessing whether the quality of the teleVA is the same as the face-to-face VA interviews. Our results suggest that the quality of teleVA responses is comparable to that of face-to-face VAs. Nonetheless, further investigation is necessary to corroborate these findings and provide more robust evidence on the equivalency of the two interview methods in additional settings prior to scaling this up nationally.

Overall, the positive outcomes and insights from the pilot study indicate that teleVAs offer promising opportunities for improving the efficiency and acceptability of conducting VA interviews, with potential benefits in different cultural contexts. Future teleVA implementations and studies will have to address the system and cultural complexities and ensure comprehensive and culturally sensitive training on how to approach and comfort respondents over the phone.

While our findings support the effectiveness of teleVAs, it is essential to acknowledge that some participants strongly prefer face-to-face VAs. The experience of one of the SAPRIN nodes suggests that the future of VA could adopt a hybrid model, combining a default teleVA approach while remaining flexible to accommodate participants’ face-to-face preferences. This model could facilitate the interviewing of older people, who may have hearing disabilities and those who are reluctant to trust someone over the phone. As far as we know, there has been no other study that has applied this hybrid model and further research will be needed to understand its potential. By striking this balance, we could optimise the benefits of teleVAs while respecting the preferences of certain participants, ensuring more inclusive and effective VA strategies.

### Limitations

A major limitation of this pilot was the identification and recruitment of NoK, due to the lack of access to current contact details. This information is routinely collected by the Department of Home Affairs during the registration of a death. However, for this pilot, these data were not available, necessitating the use of alternative recruitment methods, such as engaging with EMS and FPs. Addressing this challenge requires collaboration between sectors, including the Department of Health and the Department of Home Affairs, to effectively collect crucial information for successful teleVA implementations in the future.^6^

It is important to acknowledge the limitation that not everyone has access to smartphones which may restrict the feasibility of this approach for some individuals. Therefore, it would be important for future endeavours in this field to remain adaptive and consider the unique needs and infrastructural variations of each country and setting. By adopting a flexible and context-specific approach, we can further refine and tailor virtual assessments to ensure their optimal impact on healthcare and research practices.

Other limitations included the lack of community engagement and funding constraints which limited the number of calls to respondents. These issues are both likely to have impacted the response rate. Community engagement will be important in any future implementation of teleVA.

Additionally, the study’s comparison between telephonic and in-person VAs is limited by the differences between the two cohorts which may limit the results. While this study provides valuable insights, future research would require a comparative design to rigorously assess the differences in performance and quality between teleVAs and face-to-face VAs. Such an approach would help determine the most effective and scalable methods for capturing high-quality mortality data in diverse settings.

## Conclusion

In conclusion, VA is a critical tool to strengthen the completeness and quality of mortality statistics, especially in regions where the medical certification of the CoD data is unreliable. While face-to-face VA has been used for a long time, teleVA has been explored in recent years to reduce costs and improve efficiency. The teleVA pilot study in South Africa demonstrates that teleVAs are feasible and acceptable to both interviewers and interviewees, but identifying and recruiting NoK and training interviewers to deal with different attitudes over the phone remain challenges.

Further investigation is needed to validate our findings, particularly under ‘normal’ circumstances unaffected by the pandemic which may have inadvertently bolstered the efficacy of remote data collection. Furthermore, assessing whether teleVA maintains the same quality standards as face-to-face VA is essential. Additionally, our study highlights the necessity for comprehensive training and ongoing monitoring of teleVA interviewers to ensure the integrity and quality of VA outcomes. teleVA could present a real opportunity to reduce costs and conduct VAs efficiently.

## supplementary material

10.1136/bmjopen-2024-090708 online supplemental file 1

10.1136/bmjopen-2024-090708 online supplemental file 2

10.1136/bmjopen-2024-090708 online supplemental file 3

## Data Availability

Data are available upon reasonable request.
