# Supplementary material for: Telephonic verbal autopsies among adults in South Africa: a feasibility and acceptability pilot study
Source: BMJ Open. 2025 Feb 19;15(2):e090708. doi: 10.1136/bmjopen-2024-090708 (PMC11840908; doi:10.1136/bmjopen-2024-090708)
Supplement: online supplemental file 1 [file bmjopen-15-2-s001.docx]

## Interview Guideline (VA interviewer)

Participant ID No |__|__|__|__| Gender Male / Female

Interviewer Initials |__|__|__| Date |__|__/__|__/__|__|

District: ___________ Ward:____________ Country ______________

**Warm up [demographic & work history]**

Can I ask some details about you and your job?

1. Current Job Title ____________________________
2. Highest Educational Grade attained _________
3. VA interviewer since _______
4. Region of work_______
5. How many VA interviews have you conducted since your enrolment in the program? ______

**Feasibility**

1. How was the experience of reaching out to the families over the phone?
   1. How many times did you have to call before finding an appointment?
2. Did you find the right respondent? How did you identify he/she?
3. Are there any advantages or challenges of conducting the interview by phone?
   1. In terms of time? Costs? Quality of interview? Skills of interviewer?

**Acceptability**

1. Overall, how was it for you to conduct teleVAs compared to face-to-face VA?
2. How would you describe the communication over the phone vs face-to-face?
   1. Any important differences? What do you prefer? Why?
3. How would you describe the respondent’s attitude on the phone vs face-to-face interview?
   1. Did you see any better/worse reaction towards the questions?
4. When do you think the interviewee was more comfortable?
   1. Why?
5. How do you think any of the above influenced the quality of the interview?
6. What kind of differences do you think will appear in the results of the VAs face-to-face vs teleVA?
   1. If you had to choose in the future, which kind of interviews would you choose? Why?
   2. What would you do different if you were to do the teleVAs again?
   3. Do you have any advices for us?
