# Supplementary material for: Telephonic verbal autopsies among adults in South Africa: a feasibility and acceptability pilot study
Source: BMJ Open. 2025 Feb 19;15(2):e090708. doi: 10.1136/bmjopen-2024-090708 (PMC11840908; doi:10.1136/bmjopen-2024-090708)
Supplement: online supplemental file 2 [file bmjopen-15-2-s002.docx]

## Interview Guideline (VA respondent)

Participant ID No: |__|__|__|__| Sex: Male / Female

Interviewer Initials: |__|__|__| Date: |__|__/__|__/__|__|

Village: _________ Ward: ____________

District: ___________ Country: ______________

**Warm up [demographic & work history]**

Can I ask some details about you?

1. Highest Educational Grade attained _________
2. Current occupation
3. Relationship to the deceased?
4. When did you conduct the phone interview?

**Feasibility**

1. How has the experience of participating in this study been for you?
2. Before the phone interview, do you remember how many times the interviewer had to call before talking to you?
   1. What kind of delays did you have for this call?
3. Was there a time that was more convenient for you to conduct the interview over the phone?
4. Where did you do the phone interview?
   1. Was it difficult to find a quiet and private space?
5. How did the technology work during the interview?
   1. Any connectivity problems? Was the sound quality acceptable? Did you understand well the interviewer? Did your battery last all the time?
6. Would you describe any advantages of this method? And disadvantages?

**Acceptability**

1. How did you know you could trust the person calling over the phone?
2. How did you understand the interviewer’s questions?
3. How would you describe the communication over the phone?
4. How did you feel to discuss that type of sensitive topics over the phone?
5. If you could choose to repeat the interview, would you prefer to conduct a phone or in person VA?
   1. Why?
6. Do you have any suggestions or advice for us?
7. Any other comments?
