# Supplementary material for: Telephonic verbal autopsies among adults in South Africa: a feasibility and acceptability pilot study
Source: BMJ Open. 2025 Feb 19;15(2):e090708. doi: 10.1136/bmjopen-2024-090708 (PMC11840908; doi:10.1136/bmjopen-2024-090708)
Supplement: online supplemental file 3 [file bmjopen-15-2-s003.pdf]

Table S1: Percentage of Informative and non-informative responses in face to face VA (NCODV 2017/18) and teleVA root questions.								
**VA Question**	**NCODV face to face VA**				**teleVA**			
	**Informative**		**Non-informative**		**Informative**		**Non-informative**	
	**Yes**	**No**	**Don't know**	**Refused to answer**	**Yes**	**No**	**Don't know**	**Refused to answer**
(Id10077) Did (s)he suffer from any injury or accident that led to her/his death?	12.3%	87.5%	0.2%	0.0%	6.1%	93.9%	0.0%	0.0%
(Id10123) Did (s)he die suddenly?	23.2%	76.2%	0.6%	0.0%	56.3%	43.7%	0.0%	0.0%
(Id10125) Was there any diagnosis by a health professional of tuberculosis?	19.0%	78.3%	2.7%	0.0%	15.7%	83.8%	0.4%	0.0%
(Id10126) Was an HIV test ever positive?	24.0%	68.4%	7.6%	0.0%	9.2%	83.8%	7.0%	0.0%
(Id10127) Was there any diagnosis by a health professional of AIDS?	11.9%	80.7%	7.3%	0.1%	3.5%	90.4%	6.1%	0.0%
(Id10128) Did (s)he have a recent positive test by a health professional for malaria?	0.6%	97.7%	1.6%	0.0%	0.4%	99.6%	0.0%	0.0%
(Id10129) Did (s)he have a recent negative test by a health professional for malaria?	0.5%	97.7%	1.8%	0.0%	0.0%	99.1%	0.4%	0.4%
(Id10130) Was there any diagnosis by a health professional of dengue fever?	0.4%	98.4%	1.1%	0.0%	0.0%	100.0%	0.0%	0.0%
(Id10131) Was there any diagnosis by a health professional of measles?	0.5%	98.1%	1.3%	0.1%	0.0%	97.4%	2.6%	0.0%
(Id10132) Was there any diagnosis by a health professional of high blood pressure?	36.3%	60.7%	3.0%	0.0%	47.6%	48.5%	3.9%	0.0%
(Id10133) Was there any diagnosis by a health professional of heart disease?	9.0%	88.9%	2.0%	0.0%	21.0%	78.6%	0.4%	0.0%
(Id10134) Was there any diagnosis by a health professional of diabetes?	15.8%	82.1%	2.0%	0.0%	38.9%	59.4%	1.7%	0.0%
(Id10135) Was there any diagnosis by a health professional of asthma?	7.6%	90.7%	1.7%	0.1%	10.5%	89.5%	0.0%	0.0%
(Id10136) Was there any diagnosis by a health professional of epilepsy?	4.0%	94.6%	1.4%	0.0%	2.6%	96.9%	0.4%	0.0%
(Id10137) Was there any diagnosis by a health professional of cancer?	9.0%	89.2%	1.8%	0.0%	14.8%	84.3%	0.4%	0.4%
(Id10138) Was there any diagnosis by a health professional of Chronic Obstructive Pulmonary Disease (COPD)?	3.9%	94.7%	1.4%	0.0%	2.2%	96.5%	1.3%	0.0%
(Id10139) Was there any diagnosis by a health professional of dementia?	3.9%	94.7%	1.3%	0.1%	6.6%	93.4%	0.0%	0.0%
(Id10140) Was there any diagnosis by a health professional of depression?	5.1%	92.9%	2.0%	0.0%	4.8%	93.4%	1.7%	0.0%
(Id10141) Was there any diagnosis by a health professional of stroke?	8.7%	90.1%	1.1%	0.1%	21.0%	79.0%	0.0%	0.0%
(Id10142) Was there any diagnosis by a health professional of sickle cell disease?	0.5%	98.3%	1.2%	0.0%	0.0%	100.0%	0.0%	0.0%
(Id10143) Was there any diagnosis by a health professional of kidney disease?	7.7%	90.3%	2.0%	0.0%	10.5%	88.2%	1.3%	0.0%
(Id10144) Was there any diagnosis by a health professional of liver disease?	4.3%	93.8%	1.9%	0.0%	3.1%	96.5%	0.4%	0.0%
(Id10147) Did (s)he have a fever?	19.6%	77.5%	2.9%	0.0%	16.2%	82.5%	1.3%	0.0%
(Id10152) Did (s)he have night sweats?	30.9%	63.8%	5.3%	0.0%	19.2%	77.7%	3.1%	0.0%
(Id10153) Did (s)he have a cough?	33.4%	65.1%	1.5%	0.0%	38.9%	60.7%	0.4%	0.0%
(Id10159) Did (s)he have any difficulty breathing?	30.8%	67.4%	1.8%	0.0%	40.6%	58.5%	0.9%	0.0%
(Id10166) During the illness that led to death, did (s)he have fast breathing?	11.2%	86.6%	2.2%	0.0%	19.7%	75.1%	5.2%	0.0%
(Id10168) Did (s)he have breathlessness?	19.9%	77.3%	2.7%	0.1%	24.5%	73.4%	2.2%	0.0%
(Id10174) Did (s)he have chest pain?	25.4%	71.4%	3.1%	0.0%	19.7%	79.0%	1.3%	0.0%
(Id10181) Did (s)he have more frequent loose or liquid stools than usual?	19.7%	77.5%	2.7%	0.0%	21.8%	76.0%	2.2%	0.0%

(Id10186) At any time during the final illness was there blood in the stools?	3.6%	90.5%	5.9%	0.0%	3.9%	92.1%	3.9%	0.0%
(Id10188) Did (s)he vomit?	27.8%	70.1%	2.0%	0.0%	18.3%	81.2%	0.4%	0.0%
(Id10193) Did (s)he have any belly (abdominal) problem?	17.0%	80.9%	2.0%	0.0%	12.7%	85.2%	2.2%	0.0%
(Id10194) Did (s)he have belly (abdominal) pain?	18.5%	78.3%	3.2%	0.0%	14.0%	83.8%	2.2%	0.0%
(Id10200) Did (s)he have a more than usually protruding belly (abdomen)?	6.2%	91.7%	2.1%	0.0%	6.1%	91.7%	2.2%	0.0%
(Id10207) Did (s)he have a severe headache?	25.3%	71.8%	2.9%	0.1%	21.4%	76.0%	2.6%	0.0%
(Id10208) Did (s)he have a stiff neck during illness that led to death?	6.2%	91.4%	2.4%	0.0%	3.5%	93.4%	3.1%	0.0%
(Id10210) Did (s)he have a painful neck during the illness that led to death?	6.0%	91.1%	2.8%	0.1%	4.8%	91.3%	3.9%	0.0%
(Id10212) Did (s)he have mental confusion?	20.9%	77.1%	2.0%	0.1%	26.6%	71.6%	1.7%	0.0%
(Id10214) Was (s)he unconscious during the illness that led to death?	7.3%	90.4%	2.3%	0.0%	11.4%	86.5%	2.2%	0.0%
(Id10219) Did (s)he have convulsions?	3.1%	94.5%	2.4%	0.0%	4.8%	94.8%	0.4%	0.0%
(Id10223) Did (s)he have any urine problems?	13.2%	84.0%	2.8%	0.0%	20.1%	78.6%	1.3%	0.0%
(Id10227) Did (s)he have sores or ulcers anywhere on the body?	10.0%	88.4%	1.6%	0.0%	12.7%	86.5%	0.9%	0.0%
(Id10228) Did (s)he have sores?	10.5%	87.2%	2.3%	0.0%	15.3%	83.8%	0.9%	0.0%
(Id10230) Did (s)he have an ulcer (pit) on the foot?	3.6%	94.8%	1.6%	0.0%	5.2%	94.3%	0.4%	0.0%
(Id10233) During the illness that led to death, did (s)he have any skin rash?	3.3%	95.4%	1.3%	0.0%	4.8%	95.2%	0.0%	0.0%
(Id10237) Did (s)he ever have shingles or herpes zoster?	1.8%	96.1%	2.1%	0.0%	2.6%	94.8%	2.6%	0.0%
(Id10238) During the illness that led to death, did her/his skin flake off in patches?	4.8%	93.9%	1.3%	0.0%	5.7%	94.3%	0.0%	0.0%
(Id10241) During the illness that led to death, did (s)he bleed from anywhere?	8.1%	90.2%	1.7%	0.0%	7.4%	92.1%	0.4%	0.0%
(Id10243) Did (s)he have noticeable weight loss?	55.2%	43.5%	1.3%	0.1%	52.4%	47.6%	0.0%	0.0%
(Id10244) Was (s)he severely thin or wasted?	41.3%	57.6%	1.1%	0.0%	29.7%	70.3%	0.0%	0.0%
(Id10245) During the illness that led to death, did s/he have a whitish rash inside the mouth or on the tongue?	12.6%	85.6%	1.8%	0.1%	12.7%	85.2%	2.2%	0.0%
(Id10246) Did (s)he have stiffness of the whole body or was unable to open the mouth?	9.5%	88.9%	1.6%	0.0%	3.9%	94.3%	1.7%	0.0%
(Id10247) Did (s)he have puffiness of the face?	8.6%	89.5%	1.8%	0.1%	7.4%	91.7%	0.9%	0.0%
(Id10249) During the illness that led to death, did (s)he have swollen legs or feet?	31.6%	67.6%	0.8%	0.1%	29.3%	70.3%	0.4%	0.0%
(Id10252) Did (s)he have general puffiness all over his/her body?	6.5%	92.3%	1.2%	0.0%	2.6%	96.1%	1.3%	0.0%
(Id10253) Did (s)he have any lumps?	3.4%	94.2%	2.4%	0.0%	3.9%	94.8%	1.3%	0.0%
(Id10258) Was (s)he in any way paralysed?	11.0%	87.6%	1.5%	0.0%	11.4%	88.6%	0.0%	0.0%
(Id10261) Did (s)he have difficulty swallowing?	19.9%	78.0%	2.0%	0.0%	15.7%	81.7%	2.6%	0.0%
(Id10264) Did (s)he have pain upon swallowing?	17.3%	79.4%	3.3%	0.0%	10.9%	86.0%	3.1%	0.0%
(Id10265) Did (s)he have yellow discoloration of the eyes?	9.2%	88.7%	2.0%	0.0%	10.0%	88.2%	1.7%	0.0%
(Id10267) Did her/his hair change in color to a reddish or yellowish color?	3.0%	95.4%	1.6%	0.1%	3.1%	95.6%	1.3%	0.0%
(Id10268) Did (s)he look pale (thinning/lack of blood) or have pale palms, eyes or nail beds?	15.3%	82.7%	1.9%	0.0%	24.9%	72.9%	2.2%	0.0%

(Id10294) Did she have any swelling or lump in the breast?	2.4%	94.6%	2.9%	0.1%	4.8%	93.7%	1.6%	0.0%
(Id10295) Did she have any ulcers (pits) in the breast?	1.2%	96.2%	2.5%	0.0%	4.0%	95.2%	0.8%	0.0%
(Id10296) Did she ever have a period or menstruate?	33.0%	62.3%	4.3%	0.4%	52.4%	42.9%	4.8%	0.0%
(Id10411) Did (s)he drink alcohol?	41.7%	57.5%	0.7%	0.0%	23.6%	76.0%	0.0%	0.4%
(Id10412) Did (s)he use tobacco?	33.1%	65.7%	1.1%	0.0%	29.3%	70.3%	0.0%	0.4%
(Id10413) Did (s)he smoke tobacco (cigarette, cigar, pipe, etc.)?	35.0%	64.0%	1.0%	0.0%	29.7%	69.9%	0.0%	0.4%
(Id10418) Did (s)he receive any treatment for the illness that led to death?	62.2%	35.5%	2.3%	0.0%	60.3%	38.0%	1.3%	0.4%
(Id10450) In the final days s death, did s/he travel to a hospital or health facility?	63.8%	36.0%	0.3%	0.0%	65.1%	34.5%	0.4%	0.0%
(Id10455) Does it take more than 2 hours to get to the nearest hospital or health facility from the deceased's household?	7.1%	92.0%	0.9%	0.0%	0.4%	99.1%	0.4%	0.0%
(Id10456) In the final days before death, were there any doubts about whether medical care was needed?	19.4%	78.6%	1.9%	0.0%	17.9%	79.9%	1.7%	0.4%
(Id10457) In the final days before death, was traditional medicine used?	7.0%	91.8%	1.2%	0.0%	14.8%	84.3%	0.9%	0.0%
(Id10458) In the final days before death, did anyone use a telephone or cell phone to call for help?	71.3%	28.1%	0.6%	0.0%	73.4%	26.2%	0.4%	0.0%
(Id10459) Over the course of illness, did the total costs of care and treatment prohibit other household payments?	28.0%	71.1%	0.8%	0.0%	20.1%	78.6%	1.3%	0.0%
