# Supplementary material for: Telephonic verbal autopsies among adults in South Africa: a feasibility and acceptability pilot study
Source: BMJ Open. 2025 Feb 19;15(2):e090708. doi: 10.1136/bmjopen-2024-090708 (PMC11840908; doi:10.1136/bmjopen-2024-090708)
Supplement: Supplementary file 4 [file bmjopen-2024-090708.reviewer_comments.pdf]

PEER REVIEW HISTORY

BMJ Open publishes all reviews undertaken for accepted manuscripts. Reviewers are asked to complete a checklist review form and are provided with free text boxes to elaborate on their assessment. These free text comments are reproduced below.

ARTICLE DETAILS

Title (Provisional)

Telephonic verbal autopsies among adults in South Africa: a feasibility and acceptability pilot study

Authors

Sant Fruchtman, Carmen; Neethling, Ian; Morof, Diane; Ngwenya, Xolani; Glass, Tracy; Morden, Erna; Bradshaw, Debbie; Cobos Muñoz, Daniel; Ngobeni, Sizzy; Edwards, Anita; Kahn, Kathleen; Herbst, Kobus; Zinyakatira, Nesbert; Groenewald, Pamela

---

VERSION 1 - REVIEW

Reviewer 1

Name Maduekwe, Nnamdi Ifeanyi

Affiliation National Population Commission of Nigeria, Department of Population Studies

Date 11-Aug-2024

COI None

The manuscript (MS) represents another important attempt at proffering and analysing possible solutions to the mortality data incapacity of CRVS and other data generating systems in Low and Middle Income Countries (LMICs). At a general level the paper delves into strategies to overcome challenges with administration of data instruments in hard to reach areas, hard to reach persons and difficult situations. Specifically, it seems the study was motivated by the need to find ways around difficulties with surveillance of mortality occasioned by COVID 19 pandemic.

Like many recent studies on solutions to the mortality data challenges of LMICs it presents verbal autopsy (VA) as a pragmatic instrument and attempts to evaluate the feasibility of telephone administered VAs. The paper delves into issues related to telephone administered VAs. To its credit the MS provides a very detailed description of the procedure for implementing TeleVA using the South African experience. The study can provide useful guidance for other assessments of the viability of TeleVa and its adoption in LMICs.

To help improve the paper, I will like the authors to take note of a few general observations and some suggested corrections.

I. TeleVA Vs. Face to Face VA

Results from the paper suggest that TeleVAs perform as well as face to face interviews. However, it is noteworthy that many of the conditions necessitating the TeleVA proposal seems to have disappeared with the passing of the peak of the COVID pandemic, possibly obviating- to a large extent- the need for telephone administered VAs. This is important because, given the sensitivity of deaths and death data collection, more humane, face to face interviews are more likely to help properly calibrated data systems and well trained interviewers generate and apply the needed empathy to trace and record deaths in communities. Display of empathy and overcoming socio-cultural barriers are keys to resolving demand side issues affecting mortality data production in LMICs. This observation might help the authors in the evaluation of the terms feasibility and acceptability and moderate the interpretation of their results and recommendations.

II. Sources of Data on mortality used

Lines 148-160, Page 7, clearly describe how deaths outside health facilities were traced, namely: the WCPHDC system, emergency medical services and funeral parlours. This may be instructive in strategies to trace deaths outside health systems. As already noted, tracing and recording deaths in populations/communities not linked to established institutions remains a major challenge to comprehensive mortality data coverage in many LMICs. However, the three channels can only cover cases ‘known’ to the hospital system or the two other sources mentioned. They may not guarantee comprehensive coverage and recording of deaths outside of established institutions and channels. It might help for the authors to note this as a limitation of data sourcing in their study and a challenge that will face interventions aiming at inclusive coverage of mortality including those adopting the TeleVA option.

III. Type of VA instrument used

What was the type of VA instrument used in the study? For example, was it the WHO Standard VA package or any other? I am not sure this was explicitly mentioned or discussed.

The following are some suggested corrections and adjustments.

1. Need to define key terms

i. The terms feasibility and acceptability are key to this paper. Please define them properly and early in the MS. How did you measure feasibility and acceptability? Acceptability to whom, what, in what context. Are there standardized objective metrics (indicators) for both?

2. Imprecise use of terms:

This is a scientific journal, precision/clarity with the use of terms should be maintained. e.g.:

i. Abstract Line 26: ‘’burdensome’’?? How?

ii. Abstract Line 31 ''All interviewers were surveyed'' (why not: all interviewers were questioned or probed)

iii. Abstract Line 33: ''investigate''. Why not assess or evaluate

iv. Abstract Line 40 "TeleVA and face-to-face VA responses to root questions were similarly informative". This is not clear please, sounds subjective. Why not present in terms of statistical significance. For example: differences between TeleVA and face to face responses to root questions were not statistically significant.

v. Line 165, Page 7: ''slightly lower than expected for a telephone survey (70 – 80%) as we felt that respondents''. Please provide a reference for the expected response rate.

Some other Observations

Line 181-183 Page 7: Why the reimbursement? Is it to compensate for the air time/data expenses. Would it not have had an effect on the outcome of the interviews?

Experiences from community surveys elsewhere indicate that gifts, rewards may have some effects on interview expectation and outcomes and predispose subsequent surveys to undue expectation of reward.

**Reviewer**	**2**
**Name**	**Lam, Vienna Chichi**
**Affiliation**	**Simon Fraser University, School of Criminology**
**Date**	**24-Sep-2024**
**COI**	**No competing interests to declare.**

This was a thoughtfully designed and executed study on the feasibility of verbal autopsies. I recommend accepting this manuscript without reservations.

**Reviewer**	**3**
**Name**	**Mathew, Mary**
**Affiliation**	**Kasturba Medical College Mangalore, Pathology**
**Date**	**13-Oct-2024**
**COI**	**NONE**

Please specify the details of how the consent for the study was obtained

Reviewer **4**

Name **Chighine, Alberto**

Affiliation **Cittadella Universitaria di Monserrato, Medical Sciences And Public Health**

Date **23-Oct-2024**

COI **I declare that I have no competing interests that could influence the evaluation of this manuscript.**

Review report for manuscript bmjopen-2024-090708

I was invited to review this manuscript as the Editorial Board considered it to align with my area of expertise in forensic pathology and medicine.

The manuscript investigate the use of telephonic verbal autopsies (VAs) during two months of COVID pandemic in South Africa.

It is the reviewer understanding (lines 67-68, 91-92) that VAs are reviewed by clinical physicians who do not seems to have expertise in "proper" autopsies: this is certainly a limitation of the VA itself as physician involved in the clinical field does not have the expertise to interpret pathological findings to unravel cause, mechanism, and manner of death.

That being said the design of the experiment is characterized by a major limitation as the telephonic VAs are challenged with a different cohort of VAs conducted in person by different interviewers. Understanding the important challenges (ethical, mostly) in designing a more elegant study, this issue must figure in the limitations paragraph as the control group may differ significantly from the study group so that similarities and differencies found may not truly representative.

Results.

The authors report different answer rates according to the case sources. It is not discussed that the greater source (WCPDH) has lower response rate whereas the higher response rate was obtained from a very limited source of cases (n=7, funeral parlons). This is certainly relevant for a future implementation of the system.

Lines 285-286: As a forensic physician, I consider this to be a major limitation of VAs—how can we confidently determine the real cause of death? To illustrate one of many possible examples, a patient with leukemia might suddenly die either from cardiac complications related to chemotherapy or from a spontaneous intracerebral hemorrhage, and only a clinical or forensic autopsy could definitively establish the cause. As a result, the lower number of cases without an identified cause of death may need to be interpreted cautiously, as from a scientific perspective, the identified causes of death may often represent

presumptive and broad hypotheses. In the proposed examples, it would only be possible to state that the death could be leukemia-related, without certainty regarding the specific mechanism and cause of death cascade.

A minor comment regarding the voucher may provide valuable context for the reader in understanding how it may have influenced the respondents' decision to participate in the VAs: Was the voucher considered a significant amount for individuals from lower socioeconomic backgrounds, or was its value relatively limited?

Discussion.

According to the two goal of the study (Lines 405-407) the implementation in real-life is certainly possible once proper traditional approaches are not available (specific needs in very specific environment/period of time such as remoted regions, pandemic etc).

On the other hand, the comparative approach with face-to-face VAs may present limitations, as the control group was derived from a different dataset, which may not accurately reflect variables related to the deceased, respondents, or interviewers in the current study.

VERSION 1 - AUTHOR RESPONSE

Reviewer	Reviewer Comment	Author Response
Reviewer 4	It is the reviewer understanding (lines 67-68, 91-92) that VAs are reviewed by clinical physicians who do not seems to have expertise in "proper" autopsies: this is certainly a limitation of the VA itself as physician involved in the clinical field does not have the expertise to interpret pathological findings to unravel cause, mechanism, and manner of death.	We appreciate the reviewer's comments and acknowledge their concern regarding the expertise of clinicians reviewing verbal autopsies (VAs). However, it's important to note that VAs are a well-established method for determining cause of death, particularly in settings where traditional autopsies are not feasible.<br><br>Numerous studies have demonstrated the validity of VAs in accurately determining cause of death, often comparable to traditional autopsies. We have expanded our introduction to further highlight these findings and address the reviewer's concerns.
Reviewer 4	That being said the design of the experiment is characterized by a major limitation as the telephonic VAs are challenged with a different cohort of VAs conducted in person by different interviewers. Understanding the important challenges (ethical, mostly) in designing a more elegant study, this issue must figure in the limitations paragraph as the control group may differ significantly from the study group so that similarities and differencies	We appreciate the reviewer's observation regarding the limitations of our study design. While it would be ideal to conduct a randomized controlled trial comparing telephonic VAs to in-person VAs, ethical and practical constraints often limit such a design.<br><br>As the reviewer correctly point out, the two cohorts of VAs, conducted by different interviewers, may differ in various ways. However, our primary

	found may not truly representative.	objective was to assess the feasibility and acceptability of telephonic VAs, not to directly compare them to in-person VAs.<br><br>To assess the quality of telephonic VA responses, we compared the proportion of non-informative responses to root questions with those from a previous study. This approach, while not a direct experimental comparison, provides valuable insights into the quality of information obtained through telephonic interviews.<br><br>We have acknowledged the limitation of the different cohorts in the discussion section. We believe that our findings provide valuable information on the feasibility and acceptability of telephonic VAs, which can inform future research and implementation.
Reviewer 1	I. TeleVA Vs. Face to Face VA<br>Results from the paper suggest that TeleVAs perform as well as face to face interviews. However, it is noteworthy that many of the conditions necessitating the TeleVA proposal seems to have disappeared with the passing of the peak of the COVID pandemic, possibly obviating- to a large extent- the need for telephone administered VAs. This is important because, given the sensitivity of deaths and death data collection, more humane, face to face interviews are more likely to help properly calibrated data systems and well trained interviewers generate and apply the needed empathy to trace and record deaths in communities. Display of empathy and overcoming socio-cultural barriers are keys to resolving demand side issues affecting mortality data production in LMICs. This observation might help the authors in the evaluation of the terms feasibility and acceptability and moderate the interpretation of their results and recommendations.	Thanks for this comment, we have included in our limitations the fact that the study was conduced during COVID, to highlight the influence this may have had in response rates and also how respondents perceived teleVA. However, based on the responses during the qualitative interviews. Respondents were surprisingly positive with the experience of answering to VA over the phone.
	II. Sources of Data on mortality used<br>Lines 148-160, Page 7, clearly describe how deaths outside health facilities were traced, namely: the WCPHDC system, emergency medical services and funeral parlours. This may be instructive in strategies to trace deaths outside health systems. As already noted, tracing and recording deaths in populations/communities not	Thanks for this comment. In this study we recruited only deaths that occurred outside of the health facility.<br><br>Given the challenge to identify these deaths, we set up three mechanisms to identify deaths outside health facilities: 1) Funeral parlour, 2) Emergency Medical Services and 3) Western Cape Dept of Health linked

	linked to established institutions remains a major challenge to comprehensive mortality data coverage in many LMICs. However, the three channels can only cover cases 'known' to the hospital system or the two other sources mentioned. They may not guarantee comprehensive coverage and recording of deaths outside of established institutions and channels. It might help for the authors to note this as a limitation of data sourcing in their study and a challenge that will face interventions aiming at inclusive coverage of mortality including those adopting the TeleVA option.	with National Population Register (South Africa's civil registration database).
	III. Type of VA instrument used<br>What was the type of VA instrument used in the study? For example, was it the WHO Standard VA package or any other? I am not sure this was explicitly mentioned or discussed.	Thanks, we have added this in the text – line 155/156
	The following are some suggested corrections and adjustments.<br>1. Need to define key terms<br>i. The terms feasibility and acceptability are key to this paper. Please define them properly and early in the MS. How did you measure feasibility and acceptability? Acceptability to whom, what, in what context. Are there standardized objective metrics (indicators) for both?	Thanks, we have included these definitions – line 125-130.
	2. Imprecise use of terms:<br>This is a scientific journal, precision/clarity with the use of terms should be maintained. e.g.:<br>i. Abstract Line 26: ''burdensome''?? How?<br>ii. Abstract Line 31 ''All interviewers were surveyed'' (why not: all interviewers were questioned or probed)<br>iii. Abstract Line 33: ''investigate''. Why not assess or evaluate<br>iv. Abstract Line 40 "TeleVA and face-to-face VA responses to root questions were similarly informative". This is not clear please, sounds subjective. Why not present in terms of statistical significance. For example: differences between TeleVA and face to face responses to root questions were not statistically significant.	2.i – thanks, we changed this<br>2.ii – thanks, we changed this<br>2.iii – thanks, we changed this "investigate" for "evaluate"<br>2.iv – Similary, we have now included more details as per what we meant with informative.
	v. Line 165, Page 7: ''slightly lower than expected for a telephone survey (70 – 80%) as we felt that respondents''. Please provide a	Thanks, we have included a reference

	reference for the expected response rate.	
	Some other Observations<br><br>Line 181-183 Page 7: Why the reimbursement? Is it to compensate for the air time/data expenses. Would it not have had an effect on the outcome of the interviews?<br>Experiences from community surveys elsewhere indicate that gifts, rewards may have some effects on interview expectation and outcomes and predispose subsequent surveys to undue expectation of reward.	Thanks, we have also added here a brief explanation as per the rationale to include a reimbursement.
Reviewer 3	Please specify the details of how the consent for the study was obtained	Included line 190-191
Reviewer 4	The authors report different answer rates according to the case sources. It is not discussed that the greater source (WCPDH) has lower response rate whereas the higher response rate was obtained from a very limited source of cases (n=7, funeral parlons). This is certainly relevant for a future implementation of the system.	We thank the reviewer for highlighting the variability in response rates across different case sources. As noted, the higher response rate from funeral parlors may be attributed to the immediate contact with next-of-kin compared to the cold calling of the WCPHDC respondents and has now been added to the discussion.<br><br>While the lower response rate from WCPHDC is a limitation, it's important to consider the challenges associated with "cold-calling" and the potential impact of factors like interviewer persistence and training. As we discuss, increasing the number of call attempts significantly improved response rates.<br><br>We agree that these findings have important implications for future implementations of telephonic VA systems. We have added to the discussion that careful consideration of case source selection, interviewer training, and recruitment strategies, is needed to optimize response rates and improve the overall efficiency of the system.
Reviewer 4	Lines 285-286: As a forensic physician, I consider this to be a major limitation of VAs—how can we confidently determine the real cause of death? To illustrate one of many possible examples, a patient with leukemia might suddenly die either from cardiac complications related to chemotherapy or from a spontaneous intracerebral hemorrhage, and only a clinical or forensic autopsy could definitively establish the cause. As a result, the lower number of cases without an	We appreciate the reviewer's concern regarding the limitations of verbal autopsies (VAs) in definitively determining the cause of death. However, as indicated earlier VAs are a well-established method for estimating cause of death, particularly in settings where traditional autopsies are not feasible.<br><br>While VAs may not provide the same level of detail as a clinical or forensic autopsy, they are designed to elicit

	identified cause of death may need to be interpreted cautiously, as from a scientific perspective, the identified causes of death may often represent presumptive and broad hypotheses. In the proposed examples, it would only be possible to state that the death could be leukemia-related, without certainty regarding the specific mechanism and cause of death cascade.	information from family members and caregivers about the decedent's medical history, symptoms, and circumstances surrounding death. This information is then analyzed by trained clinicians or an algorithm to assign a probable cause of death.<br><br>It is true that VAs may not always be able to pinpoint the exact mechanism of death, especially in complex cases like the leukemia example provided. However, they can often identify broader categories of cause of death, such as infectious diseases, cardiovascular diseases, or injuries. They provide relevant cause of death information at the population level.<br><br>The lower number of cases without an identified cause of death may reflect the inherent limitations of VAs and the challenges in obtaining accurate and complete information from informants. However, it is important to note that even traditional autopsies may sometimes fail to determine a definitive cause of death.<br><br>We believe that VAs, when conducted and interpreted appropriately, can provide valuable information for public health surveillance and research. By acknowledging the limitations of the method and interpreting the findings with caution, we can use VAs to improve our understanding of mortality patterns and inform public health interventions.
Reviewer 4	A minor comment regarding the voucher may provide valuable context for the reader in understanding how it may have influenced the respondents' decision to participate in the VAs: Was the voucher considered a significant amount for individuals from lower socioeconomic backgrounds, or was its value relatively limited?	We provided a small compensation (R100, ~$6 US) for the time and inconvenience incurred. More detail has been added to the methods
Reviewer 4	According to the two goal of the study (Lines 405-407) the implementation in real-life is certainly possible once proper traditional approaches are not available (specific needs in very specific environment/period of time such as remoted regions, pandemic etc).	Yes, agreed.
Reviewer 4	On the other hand, the comparative approach with face-to-face VAs may present limitations, as the control group was derived from a different	Sure, this has been added as a limitation to the discussion section.

	dataset, which may not accurately reflect variables related to the deceased, respondents, or interviewers in the current study.	

VERSION 2 - REVIEW

Reviewer 1

Name Maduekwe, Nnamdi Ifeanyi

Affiliation National Population Commission of Nigeria, Department of Population Studies

Date 15-Jan-2025

COI

None

Reviewer 4

Name Chighine, Alberto

Affiliation Cittadella Universitaria di Monserrato, Medical Sciences And Public Health

Date 22-Jan-2025

COI

The reviewer acknowledges the overall improvement of the manuscript following revision round 1.
